# Physiological Status Prediction Based on a Novel Hybrid Intelligent Scheme

**DOI:** 10.1155/2022/4610747

**Published:** 2022-12-15

**Authors:** Na Liu, Chiyue Ma, Man Xu, Yun Ge, Dan Gan

**Affiliations:** ^1^School of Mechanical and Electrical Engineering, Shihezi University, Shihezi 832000, China; ^2^Business School, Nankai University, Tianjin 300071, China; ^3^School of Economics and Management, Hebei University of Technology, Tianjin, China

## Abstract

Physiological status plays an important role in clinical diagnosis. However, the temporal physiological data change dynamically with time, and the amount of data is large; furthermore, obtaining a complete history of data has become difficult. We propose a hybrid intelligent scheme for physiological status prediction, which can be effectively utilized to predict the physiological status of patients and provide a reference for clinical diagnosis. Our proposed scheme initially extracted the attribute information of nonlinear dynamic changes in physiological signals. The maximum discriminant feature subset was selected by employing conditional relevance mutual information feature selection. An optimal subset of features was fed into the particle swarm optimization–support vector machine classifier to perform classification. For the prediction task, the proposed hybrid intelligent scheme was tested on the Sleep Heart Health Study dataset for sleep status prediction. Experimental results demonstrate that our proposed intelligent scheme outperforms the conventional machine learning classification methods.

## 1. Introduction

In recent years, physiological status has played an important role in guiding clinical decision making [1, 2]. Medical decision makers (i.e., physicians) judge whether a patient has a disease or not usually through clinical physiological recordings [3–5]. Hence, studying physiological status-predicting methods and assisted clinical diagnosis is of practical importance. The output of physiological signals is complicated because it includes multivariate real-time monitor data and information from different physiological signals, which is huge. For this type of dynamic system, a physician using multivariate real-time physiological monitoring signals from a patient faces a great challenge to make a decision quickly and accurately. However, analyzing the previous history of physiological data trends to predict the future status of a patient has been accepted in many studies due to the difficulty in obtaining complete historical data to develop a fusion diagnosis model for predicting the physiological status of a patient [1, 6, 7]. In this study, we consider the trend in the history of physiological temporal data and the signal distribution situation to predict the future physiological status of the patient, so as to assist the physician in capturing the patient's body condition and pathological features, and make a rational diagnosis.

**Figure 1:**
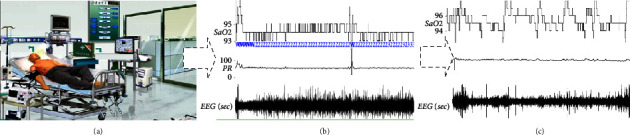
Physiological status prediction based on supervised learning. (a). Collecting the physiological status information (b). Physiological status with labeled category (c). Unlabeled category.

Sleep physiological status signal prediction is taken as an example. As can be observed in Figure 1(a), we first collect the physiological status data of patients by using the sensors, and then we rationally and effectively analyze the dynamic changes in physiological status and make predictions. Figure 1(b) indicates the history of physiological status signals of SaO_2_, PR, EEG (sec), and their labeled categories (e.g., W, 2, and 3 represent the different sleep statuses, respectively). Figure 1(c) shows the temporal unlabeled status of physiological data. Thus, a physician using labeled physiological status history cases faces a major challenge to predict unlabeled categories accurately and quickly. The most important thing is that the clinical history of physiological status data is huge, and the output of physiological signals shows certain nonlinear and nonstationary characteristics [8].

On this basis, many linear and stationary analysis methods show some limitations in dealing with physiological output signals, whereas nonlinear analysis methods have special advantages in extracting nonlinear temporal features hidden in the physiological signals [4]. The application of a nonlinear method to analyze physiological signals is helpful in identifying the potential health mechanism [9]. In this regard, our work introduced a refined composite multiscale entropy (RCMSE) method to analyze the multiple time scale data [10]. The proposed method can overcome the drawbacks of MSE, effectively reflect the dynamic changes in the time series data, and quantify the regularity of the different time scales. However, the coarse granulation features obtained by RCMSE have high dimensionality with information redundancy, which decreases the prediction accuracy and make the process time consuming. In this regard, we introduce a novel feature selection method called conditional relevance mutual information feature selection (CR-MIFS), which fully considers the dynamic changes in the selected feature with the category and overcomes the deficiency of mutual information feature selection (MIFS). We introduce a smart adaptive particle swarm optimization-support vector machine (SAPSO-SVM) method for physiological status prediction. To the best of our knowledge, SVM has been proven to be one of the most effective methods in addressing binary classification problems due to its strong generalization performance and classification precision [11–13], and the SAPSO algorithm can well optimize the parameters of the SVM classifier. Our proposed hybrid intelligent prediction scheme combines the advantages of these methods as described above so as to enhance the performance of physiological status prediction and assist clinical physicians in making correct and effective decisions.

The main contributions of our work to the field of physiological status prediction can be summarized as follows:

1. We extract the coarse granulation attributes of the physiological status information based on RCMSE, which can overcome the drawbacks of MSE, accurately estimate the complexity of the time series in different scales, and effectively reflect the dynamic changes in real-time physiological status among different time scales.
2. We introduce a novel CR-MIFS approach for coarse granulation feature selection, which can reduce the dimension of input data, improve the efficiency of predictive performance, and decrease the computational complexity to a certain extent.
3. We construct a hybrid intelligent physiological status prediction scheme that combines RCMSE for coarse granulation attribute extraction, CR-MIFS for feature selection, and SAPSO-SVM for classification. Empirical analysis verifies that our hybrid intelligent prediction scheme exhibits superior performance over other classification methods and can be accurately and effectively utilized for predicting the physiological status of patients.

The rest of this article is organized as follows: Section 2 presents a literature review on physiological status prediction.  Section 3 presents the research objectives of this study.  Section 4 proposes the framework of our hybrid intelligent prediction scheme.  Section 5 describes the empirical study of our proposed scheme, and Section 6 introduces the discussion of the hybrid scheme.  Section 7 summarizes the conclusions of this research are summarized.

## 2. Related Work

### 2.1. Physiological Status Analysis

In this section, we discuss some existing methods utilized for physiological status analysis. In 2016, Rahhal et al. [2] introduced a novel deep learning approach for electrocardiogram (ECG) signal analysis, which appropriately uses data envelopment analysis to represent the sparse features of raw ECG and introduces a deep neural network (DNN) classifier to select the most valuable ECG beats. The empirical results indicate that the proposed method is robust and computationally efficient. Dennison et al. [3] analyzed the dynamic changes in HMD and used it to predict cybersickness. The empirical results suggest that the changes in physiological measures when using an HMD to navigate a VE can be used to estimate cybersickness severity. Singh et al. [7] utilized temporal data in electronic health records (EHRs) to improve the management of chronic diseases. The empirical results show that incorporating temporal information in a patient's medical history can lead to better prediction of loss of kidney function. Nicolaou and Georgiou [4] introduced permutation entropy (PE) and SVM to detect an epileptic electroencephalogram (EEG). PE is utilized as the input feature, and SVM is applied to the segments of normal and epileptic EEG activities. The average sensitivity is 94.38%, and the average specificity is 93.23%. Yu et al. [1] constructed a novel temporal classification framework for physiological status prediction. The numerical experiment verifies the effectiveness and robustness of the classification model. Chen et al. [14] adopted multimodal feature analysis and kernel classifiers to detect the physiological signals of driving stress. The empirical analysis reveals that different levels of driving stress can be characterized by a specific set of physiological measures. Zhang et al. [6] utilized the physiological signals and reaction time to recognize different stress states. They adopted heterogeneous data for stress recognition. The SVM classifier shows good recognition performance. Chen et al. [15] proposed a system for drowsiness detection using physiological signals, which can extract evident information beyond raw signals and extract and fuse nonlinear features from EEG subbands. The empirical results reveal that the proposed method achieves high detection accuracy and extremely fast computation speed. Chen et al. [16] presented a novel method for ECG beat classification based on a combination of projected and dynamic features and adopted SVM to cluster heartbeats into one of 15 or 5 classes by using the two types of features. The empirical analysis verifies that our proposed method obtains a better performance. Elhaj et al. [17] investigated the representation ability of linear and nonlinear features and combined them to improve the classification of ECG data, which are utilized to detect arrhythmias or heart abnormalities. The empirical results show that the classification accuracy reaches 98.91%. Ullah et al. [18] proposed a system that is an ensemble of pyramidal 1D convolutional neural network (P-1D-CNN) models for epilepsy detection, achieving 99.1 ± 0.9% detection accuracy.

### 2.2. Unbalanced Data

In this section, we discuss some existing methods utilized for unbalanced physiological status analysis.

The physiological status analysis research outlined here has ignored two critical issues. The first issue is that the collected physiological signal has some nonlinear and nonstationary characteristics, and introducing an effective feature selection method to reflect the dynamic changes in the physiological signal has become important. The second issue is that the extracted physiological feature has high dimensionality, which increases computing complexity and decreases prediction performance. As shown in Table 1, most articles adopt the method of expanding unbalanced data sets for the classification tasks based on unbalanced data, which may lead to partial data distortion in the expanded data sets, thereby affecting the results. Excessive data result in a waste of time. On this basis, we construct a hybrid intelligent scheme for a physiological status prediction that can effectively extract the real-time changes in the information of physiological signals, reduce the dimensionality of the input attribute, increase the computing efficiency, and make physiological status predictions for patients.

## 3. Research Objectives

The aim of this study is to examine the performance of the proposed hybrid intelligent classification algorithm in predicting physiological status and to develop an efficient analysis framework for clinical physiological status prediction. The research objectives of this study are as follows:

1. Examine the performance of CR-MIFS for feature selection. For this research objective, we compared our proposed feature selection method with the IG, mRMR, NMIFS, and MIFS-U methods and then evaluated the average classification accuracies of different feature selection methods under the different classification methods.
2. Investigate the performance of the SAPSO-SVM classification method on physiological status prediction.
3. Design an effective hybrid physiological status predictive scheme that integrates RCMSE for coarse granulation feature extraction, CR-MIFS for feature selection, and SAPSO-SVM for classification. To verify the superior performance of our proposed method, we employ other conventional machine learning methods (CNN, SleepContextNet, XGBoost, K-NN, SVM, and SNet) for comparison and investigate their performance.

## 4. Framework of Intelligent Scheme for Physiological Status Prediction

**Figure 2:**
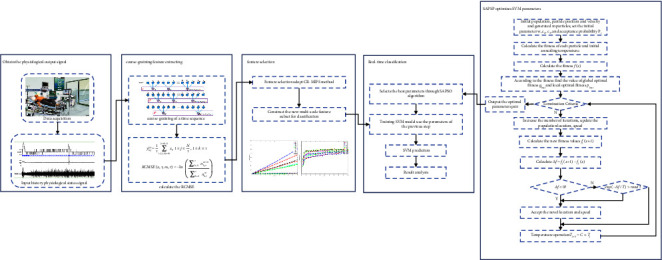
Framework of the proposed hybrid intelligent prediction scheme.

The framework of our proposed hybrid intelligent prediction scheme includes four steps, as outlined in Figure 2. In the first step, we obtain the original physiological signals from the output of the sensors. In the second module, we preprocess the original signal utilizing the RCMSE method, which can overcome the drawbacks of MSE and can effectively extract the nonlinear dynamic changes in the physiological status [10]. The RCMSE values with certain time scales are the extracted features, which reflect the complex information of temporal physiological data for different time scales and have the characteristics of high dimensionality and coarse granulation.

Although the extracted coarse granulation attribute from multiple time scales can provide abundant information for predicting physiological status, the calculation process is complex and requires excessive computer resources. In the third module, we reconstruct the feature space and select the optimal feature subset, which has the characteristics of a minimum number of attributes and maximum discrimination ability. The outstanding advantage of this feature selection is that it can reduce the dimension of the feature space, improve the physiological status-predicting efficiency, and reduce computer resources.

In the fourth module, we obtain the optimal feature subset and feed it into the SVM hybrid classifier to obtain the physiological status prediction. In our work, we adopt the radial basis function (RBF) kernel, which has been widely utilized in SVM classification [24]. To the best of our knowledge, the penalty parameters *C* and kernel function parameters *g* of the RBF kernel have some random characteristics [25], which have a great influence on SVM classification performance. In this regard, we introduce SAPSO to optimize the kernel function parameters and construct a hybrid intelligent prediction scheme to assist the physician in capturing the patient's body condition quickly and accurately. The detailed steps of our proposed hybrid intelligent prediction scheme are presented in Figure 2.

### 4.1. Dataset

Our experimental analysis was conducted on the international standard database Physiobank (Goldberger et al. [26]), which is frequently used as the benchmark dataset for the studies of physiological signal analysis papers. The experimental data are derived from the Sleep Heart Health Study (SHHS, Physionet). The SHHS is a prospective cohort study designed to investigate the relationship between sleep disordered breathing and cardiovascular disease. The Data come from 6441 individuals who were enrolled between November 1, 1995, and January 31, 1998. Each sample in this dataset includes 11 attributes: ah1: EEG, ah2: electrooculogram, ah3: electromyogram, ah4: ECG, ah5: nasal airflow, ah6: respiratory effort signals, ah7: periodic measurements of oxygen saturation (SaO_2_), ah8: periodic measurements of heart rate, ah9: annotations of sleep stages, ah10: respiratory events, and ah11: EEG arousals. In this research, we select three typical features, namely, oxygen saturation (SaO_2_), heart rate (PR), and electrocardiogram (EEG), as the input features, which are often considered the “golden standard” in the identification of sleep status [4,15,27,28]. Heart rate is abbreviated as PR with 1 Hz sampling, and the EEG sampling rate is 125 Hz. Each subject has 120^*∗*^7500 cases in 1 h, the time interval between each case is 0.004 s, and the annotations between each case is 0.5 min. On this basis, each subject includes 7500 cases, and 120 cases are found. We set the duration of the time window to 1 h, from [21 : 30] to [22 : 30]. The details of the SHHS dataset are presented in Table 2, and the input data are shown in Table 3. We show 10 cases of the input samples in Table 3. The standard deviation of input data is shown in Table 4.

### 4.2. Data Preprocessing and Feature Extraction

In this work, we introduce RCMSE for feature extraction, which is an effective method to describe the complexity and irregularity of the time series and can accurately reflect the dynamic changes in the time series [10]. We introduce the RCMSE method for physiological signal feature extraction, which can accurately reflect the abnormal fluctuations of physiological signals at a certain moment, reasonably reflect the slight change at different time scales, and has overcome the drawbacks of MSE [29]. The RCMSE algorithm includes the following three steps:

(1) For the time series of {*x*_1_, *x*_2_,…, *x*_*N*_} and the scale factor of *τ*, the coarse-grained time series can be described as follows:
  (1) $$\begin{array}{c} y_{k,j}^{\left(\tau \right)}=\frac{1}{\tau }\overset{j\tau +k-1}{\underset{i=\left(j-1\right)\tau +k}{\sum }}x_{i},1\leq j\leq \frac{N}{\tau },1\leq k\leq \tau \text{.} \end{array}$$
(2) For the scale factor of *τ*, the number of matched vector pairs *n*_*k*,*τ*_^*m*+1^ and *n*_*k*,*τ*_^*m*^ is computed, where *n*_*k*,*τ*_^*m*^ represents the total number of *m*-dimensional matched vector pairs and is computed from the *k* th coarse-grained time series at a scale factor *τ*.
(3) RCMSE is then defined as follows:

(2) $$\begin{array}{c} \text{RCMSE}\left(x,\tau ,m,r\right)=-In\left(\frac{\sum_{k=1}^{\tau }n_{k,\tau }^{m+1}}{\sum_{k=1}^{\tau }n_{k,\tau }^{m}}\right)\text{.} \end{array}$$

RCMSE can qualify the average uncertainty and evaluate the complexity of the physiological attribute, where *x* represents the time series {*x*_1_, *x*_2_,…, *x*_*N*_}, *m* represents the dimension, *τ* represents the scale factor, and *r* represents the vector capacity. Large RCMSE values indicate that the information and complexity of the temporal time series data are great and the final results are small. By contrast, a small RCMSE value indicates that the temporal data are greatly ordered and the final results are great [1].

### 4.3. Reconstructed Feature Space and Feature Selection

The physiological status includes multivariate dimensional data, and each dimension of the feature includes different time scales; thus, we should reconstruct the feature space and establish a convenient feature retrieval method. For the time scale of {*e*_1_, *e*_2_, *e*_3_,…, *e*_*n*_} and the feature space of *F*={*f*_1_^*p*_1_^, *f*_2_^*p*_1_^,…, *f*_*e*_1_−1_^*p*_1_^, *f*_*e*_1__^*p*_1_^, *f*_1_^*p*_2_^, *f*_2_^*p*_2_^,…, *f*_*e*_2_−1_^*p*_2_^, *f*_*e*_2__^*p*_2_^,…, *f*_1_^*p*_*m*_^, *f*_2_^*p*_*m*_^,…, *f*_*e*_*n*__^*p*_*m*_^}, where *P*={*p*_1_, *p*_2_,…, *p*_*m*_} is the coarse granulation attributes, which have the same scale of multiple attributes and the same attribute of different scales, the reconstructed feature space based on these features is *F*={*e*_*i*_|*i*=1,2,…, *n*}, where *e*_*i*_ is the new feature index, and any two elements in the feature space can be expressed as *f*_*e*_*i*__ ∈ *F*, *f*_*e*_*j*__ ∈ *F*, where *e*_*i*_ and *e*_*j*_ are their index of the element, and *i* ≠ *j*. Suppose we have physiological status labeled dataset *D*, which comprises *ε* samples with *p*_*m*_ features, that is, *D*={(**x**_**i**_^(**p**)^, **y**_**i**_^(**p**)^)}, where **x**_**i**_^(**p**)^=(x_1_^(p)^,…, x_N_^(p)^) ^*T*^ is the N-dimensional reconstructed feature vector of the *p*th sample, and **y**_**i**_^(**p**)^ is the *p*th sample's class label. We can transform the complex real-time physiological status of multiscale input features into a simple decision table with the reconstructed feature vector of its corresponding category through the reconstructed multiscale feature space. However, the feature vectors obtained by the RCMSE method for feature extraction have high dimensionality with information redundancy, which decreases the prediction accuracy and makes the process time-consuming. In this regard, feature selection has become necessary, which can reduce the dimension of the reconstructed feature space, decrease the calculation complexity, and improve the classification efficiency. Mutual information (MI) has been widely utilized for feature selection, which can quantify the information between different attributes and is a good indicator of the correlation between multiscale features [30–32]. In Shannon's information theory [19], the reconstructed coarse granulation feature is regarded as the input, and the information entropy of the reconstructed feature *f*_*τ*=*i*_^*p*_*j*_^ (where *f*_*τ*=*i*_^*p*_*j*_^ is the value of *i* th time scale under the *p*_*j*_ th feature) can be defined as follows:

(3) $$\begin{array}{c} H\left(f_{i}^{p_{j}}\right)=-\underset{f_{i}\in S}{\sum }p\left(f_{i}^{p_{i}}\right)\log\;p\left(f_{i}^{p_{j}}\right)\text{.} \end{array}$$

For two multiscale features of *f*_*i*_^*p*_*j*_^ and *f*_*k*_^*p*_*j*_^, the joint entropy of *f*_*i*_^*p*_*j*_^ and *f*_*k*_^*p*_*j*_^ is defined as follows:

(4) $$\begin{array}{c} H\left(f_{i}^{p_{j}},f_{k}^{p_{j}}\right)=-\underset{f_{i}^{p_{j}}\in S_{1}}{\sum }\underset{f_{k}^{p_{j}}\in S_{2}}{\sum }p\left(f_{i}^{p_{j}},f_{k}^{p_{j}}\right)\log\;p\left(f_{i}^{p_{j}},f_{k}^{p_{j}}\right)\text{.} \end{array}$$

During the process of feature selection, some of the features are determined and others are not. We define conditional entropy as the measurement of attribute uncertainty.

(5) $$\begin{array}{c} H\left(\left.f_{i}^{p_{j}}\right|f_{k}^{p_{j}}\right)=-\underset{f_{i}^{p_{j}}\in S_{1}}{\sum }\underset{f_{k}^{p_{j}}\in S_{2}}{\sum }p\left(f_{i}^{p_{j}},f_{k}^{p_{j}}\right)\log\;p\left(\left.f_{i}^{p_{j}}\right|f_{k}^{p_{j}}\right)\text{.} \end{array}$$

where *p*(*f*_*i*_^*p*_*j*_^|*f*_*k*_^*p*_*j*_^) is the posterior probability of attribute *f*_*k*_^*p*_*j*_^ given attribute *f*_*i*_^*p*_*j*_^. The conditional entropy indicates the amount of uncertainty left in attribute *f*_*k*_^*p*_*j*_^ if attribute *f*_*i*_^*p*_*j*_^ is introduced. The relationship between joint entropy and conditional entropy can be defined as follows:

(6) $$\begin{array}{c} H\left(f_{i}^{p_{j}},f_{k}^{p_{j}}\right)=H\left(f_{i}^{p_{j}},f_{k}^{p_{j}}\right)+H\left(\left.f_{i}^{p_{j}}\right|f_{k}^{p_{j}}\right)\text{.} \end{array}$$

The MI between two attributes can be defined as follows:

(7) $$\begin{array}{c} I\left(f_{i}^{p_{j}},f_{k}^{p_{j}}\right)=\underset{f_{i}^{p_{j}}\in S_{1}}{\sum }\underset{f_{k}^{p_{j}}\in S_{2}}{\sum }p\left(f_{i}^{p_{j}},f_{k}^{p_{j}}\right)\log\frac{p\left(f_{i}^{p_{j}},f_{k}^{p_{j}}\right)}{p\left(f_{i}^{p_{j}}\right)\cdot p\left(f_{k}^{p_{j}}\right)}\text{.} \end{array}$$

MI can be expressed in the form of entropy as follows:

(8) $$\begin{array}{c} I\left(f_{i}^{p_{j}},f_{k}^{p_{j}}\right)=H\left(f_{i}^{p_{j}}\right)+H\left(f_{k}^{p_{j}}\right)-H\left(f_{i}^{p_{j}},f_{k}^{p_{j}}\right)\text{,} \end{array}$$

(9) $$\begin{array}{c} \max\left\{\underset{\text{relevance}}{\underbrace{\underset{f_{i}^{P_{m}}\in S}{\sum }I\left(f_{i}^{P_{m}};C\right)}}-\right.\left.\beta \underset{\text{redundancy}}{\underbrace{\underset{f_{i}^{p_{m}}\in S}{\sum }\underset{f_{k}^{p_{z}}\in S}{\sum }I\left(f_{i}^{p_{j}},f_{k}^{p_{j}}\right)}}\right\}\text{.} \end{array}$$

Information entropy has been utilized to solve the problem of quantifying information. The higher the value of information entropy, the greater the randomness of the time series. MI has been widely utilized for feature selection because it can effectively quantify the correlation of the attribute and is insensitive to noise or outlier data [33]. If the value of MI between two attributes is large, then the correlation of the attributes is closely related. If MI is zero, then the two multiscale attributes are completely unrelated. Previous studies proposed many types of MI feature selection algorithms, such as mRMR [34], MIFS [35], MECY-FS [30], MIFS-U [36], and NMIFS [37]. However, these methods have some drawbacks. The first drawback is that they combine feature relevance and redundancy measures for feature selection, utilize a parameter to control the trade-off between feature relevance and redundancy, which is uncertainty, and influence the optimal feature subset, as shown in formula (9). The second drawback is that they only consider the candidate feature relevancy and class, and ignore the selected feature when calculating feature relevance. However, the relevancy between the candidate feature and class is dynamically changed with the addition of the selected feature [32, 38]. In this regard, we fully consider the conditional feature relevance and uncertainty parameter and adopt a novel feature selection method called CR-MIFS, which considers the dynamic information of the selected feature with the class. In accordance with the mRMR criteria [34], set *β* is equal to the inverse of the number of selected features.

(10) $$\begin{array}{c} \max\left\{\underset{\text{relevance}}{\underbrace{\underset{f_{i}^{P_{m}}\in S^{\prime }}{\sum }I\left(f_{i}^{P_{m}};C\right)}}-\right.\left.\frac{1}{\left|S\right|}\underset{\text{redundancy}}{\underbrace{\underset{f_{i}^{p_{m}}\in S^{\prime }}{\sum }\underset{f_{k}^{p_{z}}\in S}{\sum }I\left(f_{k}^{p_{z}},f_{i}^{p_{m}}\right)}}\right\}\text{,} \end{array}$$

where *f*_*i*_^*p*_*m*_^ is the candidate feature, and *f*_*k*_^*p*_*z*_^ is the selected feature. *S*′ is the candidate feature set, and *S* represents the selected feature set. In (10), which ignores the relevance of the selected feature and class, the relevance dynamically changes with the addition of the selected feature. Therefore, we employ the CR-MIFS method, as shown in the following equation:

(11) $$\begin{array}{c} \max\left\{\underset{\text{relevance}}{\underbrace{\underset{f_{i}^{P_{m}}\in S^{\prime }}{\sum }I\left(f_{i}^{P_{m}};\left.C\right|f_{k}^{p_{z}}\right)}}-\right.\left.\frac{1}{\left|S\right|}\underset{\text{redundancy}}{\underbrace{\underset{f_{i}^{p_{m}}\in S^{\prime }}{\sum }\underset{f_{k}^{p_{z}}\in S}{\sum }I\left(f_{k}^{p_{z}},f_{i}^{p_{m}}\right)}}\right\}\text{,} \end{array}$$

where we consider the selected feature and calculate the mutual information of the candidate feature and class when given the selected feature. The pseudocode of CR-MIFS is presented in Algorithm 1.

In Algorithm 1, *F* is the reconstructed coarse granulation features, including different time scales and physiological attributes. The category label *C* reflects the different physiological statuses corresponding to different coarse granulation attributes. Maxs is a variable that stores the variable of the feature of maximal conditional relevance and minimal redundancy. *f*_*i*_^*p*_*j*_^ is the selected feature.

### 4.4. Physiological Status Prediction by the SAPSO-SVM Algorithm

**Figure 3:**
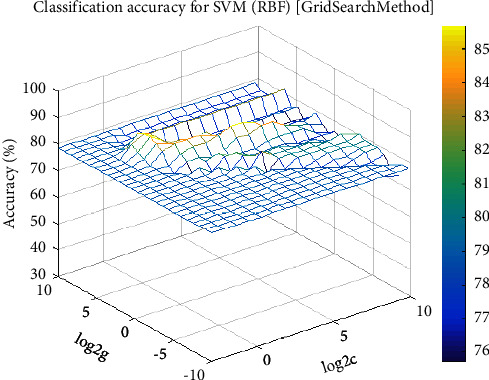
Classification accuracies achieved by the SVM classifier with RBF kernel.

An intelligent pattern classification method is required to automatically fulfill the physiological status predictions after obtaining the features to represent the primary physiological information of dynamically changed physiological signals. In this work, we introduce SVM for classification performance measurement. To the best of our knowledge, SVM utilizes convex quadratic programming, which provides only the global minimum. Thus, it avoids being trapped in local minima [25, 39]. We utilize the LIBSVM package, which supports two-class and multiclass classification [40]. However, some improvements to SVM are still required when we perform the classification tasks. The penalty parameter C and the kernel function parameter *g* have some random characteristics, which remarkably influence the classification accuracy.  Figure 3 describes the classification accuracy result for the SVM classifier with RBF kernel in the SHHS dataset (Physionet). In our empirical study, we perform a fivefold cross-validation on the 70%–20% training–testing partition of the dataset and set the variation range of parameter C from 2^(−10) to 2^(10). The variation range of parameter g is 2^(−10) to 2^(10), and the step of average classification accuracy is 0.2.

In this empirical study, we investigate the classification performance of the SVM classifier under the different parameter settings. The traditional searching approaches, such as the gradient descent method [41] and Tabu search method [42], are vulnerable to falling into the local optimum and cannot output the global optimal solution. Therefore, we select the particle swarm optimization (PSO) algorithm [43], which is based on the simulation of the social behavior of organisms. PSO has certain outstanding merits, such as a simple computational process, easy implementation, less parameters, and fast convergence. PSO-SVM has a better performance than other methods [44], such as genetic algorithm, information gain, and relief algorithm. However, the PSO algorithm can easily fall into the local optimum and undergo premature convergence in the global search process. The effect of random oscillation is reduced during the later stage of convergence [45]. Motivated by this deficiency, we introduce a simulated annealing (SA) algorithm to modify PSO [46] by taking the parameters *C* and *g* of the RBF kernel function as the position of particles. When PSO completes updating the position of particles and calculating the new fitness function, the new fitness function is taken as the objective function of SA, and the difference between the fitness value of particles in the new position and the fitness value of the historical position is calculated. If the difference meets the judgment criteria, then the position and speed of current particles are accepted; otherwise, they are accepted with probability exp (−Δ*f*/*T*). The annealing temperature is adjusted, the cycle standard is determined whether it is achieved, and the best location of the particles is outputted. The hybrid algorithm can jump out from the local optimum region and dynamically adjust the annealing temperature. With the decrease in temperature, the particles are in a low-energy state and converge to a global optimal solution. The specific steps of the SAPSO-SVM algorithm are shown in Figure 2.

## 5. Experimental Analysis

A comprehensive numerical experiment was conducted on the MATLAB 2016a platform to examine the predictive performance of the proposed intelligent prediction scheme on physiological status prediction. The performance parameters of the executing host are Windows 10 with an Intel (R) Core(TM) i5-1135g7 CPU at 2.40 GHz, X64, and 8 GB (RAM).

### 5.1. Evaluation Measure

The average classification accuracy (ACC), F1-score, and kappa coefficient are utilized as the evaluation measures to evaluate the predictive performance of our proposed method. ACC is a widely utilized measure in the performance evaluation of classification algorithms and is the ratio of true positives and true negatives to the total number of instances. The ACC calculation formula is given as follows:

(12) $$\begin{array}{c} \text{ACC}=\frac{\text{TN}+\text{TP}}{\text{TP}+\text{FP}+\text{FN}+\text{TN}}\text{,} \end{array}$$

where TP is the number of cases correctly classified to sleep status category C1; FP is the number of cases belonging to sleep status category C2 misclassified to category C1; TN is the number of cases correctly classified to sleep status category C2; FN is the number of cases belonging to sleep status category C1 misclassified to category C2. The evaluation methods are based on the confusion matrix, as shown in Table 5.

F1-score is an index used to measure the accuracy of the dichotomous (or multitask dichotomous) model in statistics. The calculation formula is given as follows:

(13) $$\begin{array}{c} F1=\frac{2\times \text{pre}\times \text{rec}}{\text{pre}+\text{rec}}\text{,} \end{array}$$

where pre denotes the precision, and rec represents the recall rate.

Kappa coefficient is an indicator for the consistency test. The calculation formula is given as follows:

(14) $$\begin{array}{c} \text{Kappa}=\frac{\text{ACC}-p_{c}}{1-p_{c}}\text{,} \end{array}$$

where *p*_*c*_ is the proportion of agreements expected by chance.

### 5.2. Experimental Procedure

#### 5.2.1. Data Preprocessing and Feature Extraction

The SHHS dataset includes 120 subjects, and we set the time series of {X1, X2, X3,…, X120}, where each subject includes 7500 cases, and the time slice is set to 0.5 min. For example, given that the time stamp at 0.5 min was 7501, the temporal data of X2 are {7501 : 15,000, X2}. Table 3 shows 10 cases of the input data. Each case includes the 3D input feature {SaO2, PR, EEG} and the annotations of the physiological status, and each feature is demonstrated by the first observation, the last on (7500th), and the minimum and maximum values. The decision state of each row in the table is identified as follows: C1, which represents the sleep status of being awake or waiting to sleep; C2, which depicts the various stages of sleeping. As shown in Table 3, the real-time physiological status of the patient can be reflected by the input features. In this work, we select SaO2 (%), PR (BPM), and EEG (uV) as our input features. The history of the physiological status of the output signals has some nonlinear characteristics, and the physiological status information of the patients must be extracted. Therefore, we adopt the RCMSE algorithm to extract the physiological output signal, which can reflect the dynamic changes in the physiological status and accurately obtain the complex information of the time series.

**Figure 4:**
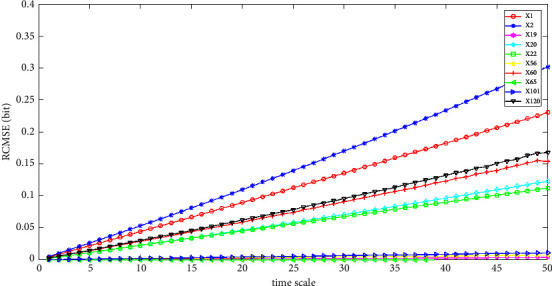
RCMSE of the temporal feature SaO2.

**Figure 5:**
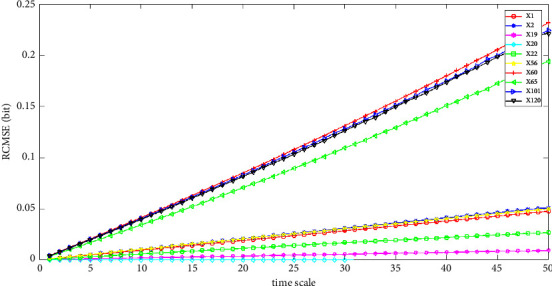
RCMSE of the temporal feature PR.

**Figure 6:**
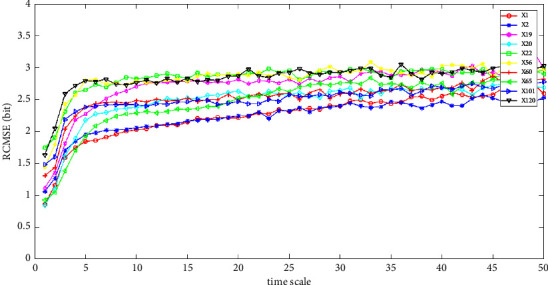
RCMSE of the temporal feature EEG.

The feature extraction results are shown in Figures 4–6. The temporal features of SaO_2_, RP, and EEG of the 10 cases in the SHHS dataset are shown. We calculated the RCMSE values from a scale of 1 to 50, and the SampEn was calculated with *m*=2 and *r*=0.2 × *σ*, where *σ* denotes the standard deviation of the original time series. Here, we set the base of the logarithm to two, so the unit of the entropy is a bit. From Figures 4–5, the value of the RCMSE curve ascends gradually with the increase in the number of time scales. As shown in the results presented in Figure 6, the results of the RCMSE curve change quickly when the time scales are smaller than 5, whereas the RCMSE becomes gentle when the time scale is greater than 5. To the best of our knowledge, the larger the value of the RCMSE, the less we believe in the final results [1]. Therefore, the more complex the time series data, the less we believe in the final results with the increase in the time scale.

#### 5.2.2. Reconstructed Feature Space and Feature Selection

In this empirical study, we select the time scale of *τ*=1,2,3,5; we then obtain a reconstructed feature space of *F*={*F*_1_, *F*_2_, *F*_3_, *F*_5_}, as shown in Table 6. We obtain different reconstructed feature subsets that belong to different physiological attributes in accordance with the reconstructed feature space. As shown in Table 6, feature subsets {f1, f2, f3, f4}, {f5, f6, f7, f8}, and {f9, f10, f11, f12} represent the reconstructed physiological features of SaO2(%), PR(BPM), and EEG(uV), respectively. We introduce the RCMSE method to extract the coarse-grained information of the physiological signal, and the results are presented in Table 7. Here, we only presented 10 cases of information values (bit) of the SHHS samples.

**Figure 7:**
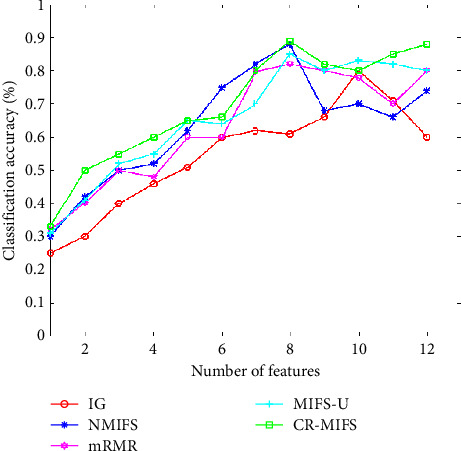
Average classification accuracy achieved with SVM, NB, and 3NN in the SHHS dataset.

When we select SaO2, PR, and EEG as the input 3D features, we obtain 4095 (24 × 24 × 24 − 1) types of feature space combinations, although some of the reconstructed multiscale feature space may not represent the complete information of the original feature set. In this regard, we adopt the CR-MIFS method for feature selection, which can select the optimal feature subset with the same discrimination ability as the original feature set and can fully consider the relevance between the candidate feature and class when given the selected feature. The classification performance of our proposed feature selection is compared with IG, mRMR, NMIFS, and MIFS-U on the SHHS dataset.  Table 8 shows the order of selected features for the IG, NIMIFS, mRMR, MIFS-U, and CR-MIFS methods. To evaluate the performance of the classification accuracy against the number of features, we introduce three different classifiers: SVM with RBF kernel, Naïve-Bayes (NB), and three-nearest Neighbors (3NN), which are used to evaluate the classification accuracies in the SHHS dataset. As shown in Figure 7, the number of feature *n* on the *X*-axis represents the first selected feature by different classifiers, and the *Y*-axis represents the average accuracy for the first selected *n* features. We set the number of multiscale selected features from 1 to 12 and employ fivefold cross-validation to obtain the highest classification accuracy through different classifiers. We calculate the average classification accuracies in accordance with the three highest accuracies, as described in Figure 7.

Figure 7 indicates the average classification accuracy achieved with SVM (RBF), NB, and 3NN based on different feature selection algorithms. As observed in Figure 7, the classification accuracy curve ascends gradually with an increase in the number of the selected features when we select the first eight features. The average classification accuracy is 88.91% with the CR-MIFS algorithm. During this process, we obtain the optimal feature subset *F* = {f2, f1, f3, f4, f5, f6, f9, f12}. The CR-MIFS method outperforms the IG, NMIFS, mRMR, and MIFS-U methods in the SHHS dataset.

#### 5.2.3. Physiological Status Prediction

We compare our proposed scheme with five conventional machine learning classification methods (CNN, SleepContextNet, XGBoost, K-NN and SVM, SNet) to verify its performance. We quote the results in previous papers and adopt accuracy, F1-score, and kappa coefficient as the evaluation standard of the model.  Table 9 shows the comparison results of accuracy, F1-score, and kappa coefficient of the different classification methods in the PhysioNet dataset. In [47], Arnaud et al. utilized CNN to predict five sleep stages, and the accuracy, F1-score, and kappa coefficient are 87%, 0.78, and 0.81, respectively. In [48], Caihong et al. designed a sleep staging network named SleepContextNet for sleep stage sequence. The accuracy, F1-score, and kappa coefficient are 86.4%, 0.8, and 0.81, respectively. In [49], Cong et al. proposed a classification model with the XGBoost algorithm and tested it using fivefold cross-validation on three different databases. In the tasks of 4-class and 5-class sleep staging, the proposed method achieved an accuracy of 87.5% and 85.8% in the SHHS database, respectively, and the kappa coefficient is 0.79 and 0.81, respectively. In [50], Seda et al. utilized Alex-Net and VGG-16 for feature extraction, K-NN, and SVM for classification, and the accuracy and F1-score are 92.78% and 0.93, respectively. In [51], Kuo et al. proposed SNet, which achieves the highest accuracy in single CNN for EEG spectrogram classification, and the accuracy, F1-score, and kappa coefficient are 93.80%, 0.79, and 0.86, respectively. The accuracy, F1-score, and kappa coefficient of our method are 97.15%, 0.94, and 0.88, respectively, which are higher than those of other methods.

#### 5.2.4. Results

We selected SaO_2_, PR, and ECG as input features. We extracted the dynamic features using the RCMSE method, utilized the CR-MIFS method for feature selection, and applied the SAPSO-SVM classifier for physiological state classification. In the experiment, the accuracy, F1-score, and kappa coefficient of our method reached 97.15%, 0.94, and 0.88, respectively. To obtain the final classification results, we use five conventional machine learning classification methods (CNN, SleepContextNet, XGBoost, K-NN and SVM, SNet) as comparison methods, and the results are presented in Table 9. In accordance with Table 9, the best (highest) results obtained by different methods verify the excellent performance of our proposed method. To elaborate further, we design three issues in our empirical study.

The first design issue of our study is to investigate the performance of our proposed CR-MIFS method for feature selection. In this regard, we set IG, NMIFS, mRMR, and MIFS-U as the comparison methods and obtain the classification results of different feature selection methods based on SVM, NB, and 3NN classifiers, as shown in the results in Figure 7.

The second design issue of our study is to verify the superiority of the SAPSO algorithm in optimizing the parameters of the SVM classifier.

The third design issue of our empirical study is to illustrate the superior predictive performance of our proposed scheme. To this end, five traditional machine learning classification methods are considered, and we design a set of comparative experiments, where their results are shown in Table 9. The proposed physiological status prediction scheme yields the highest predictive performance compared with other methods.

## 6. Discussion

In this section, we provide a discussion of the performance of our proposed hybrid intelligent prediction scheme. As mentioned previously, our proposed hybrid intelligent scheme includes different data processing steps.

The first step is coarse granulation feature extraction. We employ the RCMSE method to extract the coarse granulation time series data, which overcomes the deficiencies of the MSE method and can effectively reflect dynamic changes in physiological status accurately. In our empirical study, we introduce the SHHS dataset as a benchmark dataset, and the RCMSE results are shown in Figures.  4–6. From the trend of these curves, the value of RCMSE ascends gradually with the increase in the time scale, and the more complex the time series data, the less we believe in our final results. On this basis, we select *τ*=1,2,3,5 as four time scales in our empirical analysis and reconstruct our feature space, as shown in Table 7. We select the physiological features of SaO_2_ (%), PR (BPM), and EEG(uV) as the input 3D features to obtain 4095 types of feature combinations, although some of the reconstructed feature space may not represent the complete information of the original feature set. In the next step, we employ the CR-MIFS method for feature selection, and we compare it with IG, mRMR, NMIFS, and MIFS-U in the SHHS dataset to evaluate its performance. The results are presented in Figure 7. The results show that our CR-MIFS method outperforms the other feature selection methods because it achieves 88.91% classification accuracy. The last step is to rationally predict the physiological status. In our empirical analysis, we employ SVM for physiological status prediction. To the best of our knowledge, the penalty parameter C and the kernel function parameter g have a remarkable influence on the classification accuracy whenever we apply SVM for classification (as shown in Figure 3). Motivated by this deficiency, we introduce the SAPSO method to optimize the SVM parameters.

Regarding the hybrid scheme to be utilized in physiological status prediction, we select five conventional machine learning classification methods (CNN, SleepContextNet, XGBoost, K-NN, SVM, and SNet) as the comparison methods, and the results are presented in Table 9. The results indicate that our proposed scheme has a superior performance to other conventional classification methods, and its prediction accuracy, F1 score, and kappa coefficient are 97.15%, 0.94, and 0.88, respectively. Our research has a number of practical implications. The extraction of coarse-grained features and the selection of compact attribute space in our work have become critical issues in developing an intelligent scheme, which is of great importance in physiological status prediction. Our proposed intelligent scheme can be utilized as a decision support tool to assist disease diagnosis in clinics.

## 7. Conclusions

This work proposes a hybrid intelligent prediction scheme, which fuses the RCMSE method for coarse granulation feature extraction, the CR-MIFS method for feature selection, and SAPSO-SVM for physiological status prediction. The performance of our proposed scheme is tested in the SHHS dataset and compared with five conventional machine learning classification methods, namely, CNN, SleepContextNet, XGBoost, K-NN, SVM, and SNet. The empirical results verify that our designed hybrid intelligent scheme shows outstanding performance in physiological status prediction. The main objective of this work is to combine the advantages of these methods so as to enhance the performance of our physiological status prediction and assist clinical physicians in making correct and effective decisions.

## Figures and Tables

**Algorithm 1:**
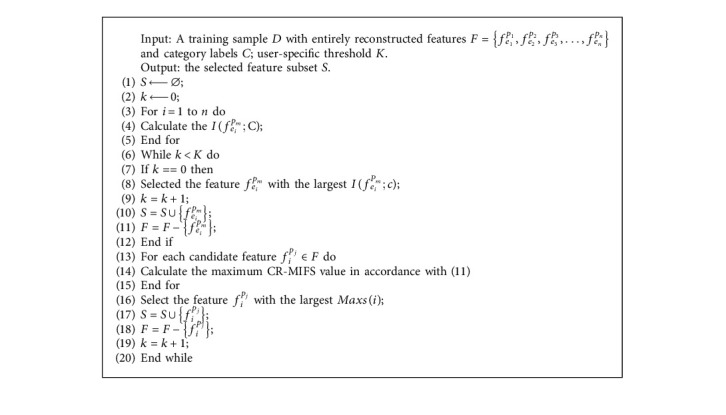
CR-MIFS

**Table 1:** Relate work of unbalanced data.

Author	Title	Method	Shortcoming
Riskyana et al. [19]	Generative adversarial networks for unbalanced fetal heart rate signal classification	They utilized time series generative adversarial networks (TSGAN) to solve data imbalance in the fetal heart rate (FHR) signal and generate more data and better classification performance.	Data enhancement is used to process unbalanced data, resulting in huge data volume and increased computing burden
Xinyu Luo et al. [20]	Multi-classification of arrhythmias using an HCRNet on imbalanced ECG datasets	They developed a new, more robust network model named hybrid convolutional recurrent neural network (HCRNet) for the time-series signal of ECG.	This work needs a large amount of data and the time cost of the training phase and the model's training by using 10-fold cross-validation is very time-consuming and makes a demand for use of high-tech computers.
Georgios et al. [21]	Automated atrial fibrillation detection using a hybrid CNN-LSTM network on imbalanced ECG datasets	They propose a novel hybrid neural model utilizing focal loss, an improved version of cross-entropy loss, to deal with training data imbalance. ECG features initially extracted via a convolutional neural network (CNN) are input to a long short-term memory (LSTM) model for temporal dynamics memorization and thus, more accurate classification into the four ECG rhythm types	the proposed network was tested only on four beat types, classes AFL and J represent only an extremely small percentage of the total dataset and the model's training by using 10-fold cross validation is very time consuming and makes a demand for use of high-tech computers.
Tianyu et al. [22]	A hybrid machine learning approach to cerebral stroke prediction based on an imbalanced medical dataset	Firstly, random forest regression is adopted to impute missing values before classification. Secondly, an automated hyperparameter optimization(AutoHPO) based on a deep neural network(DNN) is applied to stroke prediction on an imbalanced dataset.	Data enhancement is used to process unbalanced data, resulting in huge data volume and increased computing burden
Chaofan et al. [23]	Classification of imbalanced electrocardiosignal data using convolutional neural network	An improved data augmentation method based on variational auto-encoder (VAE) and auxiliary classifier generative adversarial network (ACGAN) is implemented to address the difficulties resulting from the imbalanced dataset. Based on the augmented dataset, convolutional neural network (CNN) classifiers are employed to automatically recognize arrhythmias using two-dimensional ECG images.	The main disadvantage of this study is the time cost of training deep models. The VAE and ACGAN need to be trained separately, which will cost a lot of time and computation. Also, due to the complicated nature of deep models, the proposed algorithm needs sophisticated hardware to realize the arrhythmia detection function.

**Table 2:** Details of the SHHS dataset.

Characteristics	Attribute	Tasks	Subjects	Instance of each subject	Number of attributes
Temporal multivariate	Integer	Classification	5804	120^*∗*^7500	11

**Table 3:** Ten cases of the input samples.

Cases	Slices (min)	Features												Label
		SaO_2_ (%)				PR (BPM)				EEG (uV)				
		1	min	Max	7500	1	min	Max	7500	1	min	Max	7500	
X1	0.00	96.011	94.013	97.01	94.013	67.265	67.265	82.238	78.242	−3.922	−77.451	98.039	18,627	C1
X2	0.50	94.013	94.013	96.011	95.012	78.242	39.316	78.242	50.305	19.608	−114.706	108.824	7.843	C1
X19	9.00	95.012	94.013	95.012	94.013	66.266	64.268	66.266	64.268	12.745	−39.216	65.686	−6.863	C2
X20	9.50	94.013	94.013	94.013	94.013	64.268	64.268	67.265	65.267	−11.765	−90.196	99.02	−4.902	C1
X22	10.5	95.012	95.012	98.009	98.009	71.261	62.282	71.261	65.267	−3.922	−32.353	44.118	−3.922	C1
X56	27.5	96.011	95.012	96.011	95.012	70.262	63.268	70.262	63.268	13.725	−44.118	36.275	−18.627	C2
X60	30.5	95.012	94.013	96.011	96.011	60.284	58.285	65.267	58.285	13.725	−48.039	67.647	−13.725	C2
X65	32	95.012	95.012	95.012	95.012	59.284	57.286	60.284	59.284	36.275	−120.588	124.51	18.627	C2
X101	50	96.011	95.012	96.011	95.012	63.268	61.283	66.266	66.266	10.784	−80.392	78.431	-32.353	C1
X120	59.5	98.009	96.011	98.009	96.011	65.267	63.268	67.265	63.268	16.667	−43.137	46.078	2.941	C1

**Table 4:** Standard deviation.

Features	SaO_2_				PR (BPM)				EEG (uV)			
	1	min	Max	7500	1	min	Max	7500	1	min	Max	7500
Standard deviation	1.229	18.964	1.183	1.213	5.26	13.139	5.741	5.228	17.031	31.429	33.266	20.016

**Table 5:** Confusion matrix.

	Predicted category C1	Predicted category C2
Actual category C1	TP	FN
Actual category C2	FP	TN

**Table 6:** Reconstructed feature space.

Sets	F1 (*τ*=1)	F2 (*τ*=2)	F3 (*τ*=3)	F5 (*τ*=5)
F	{*f*_1_, *f*_5_, *f*_9_}	{*f*_2_, *f*_6_, *f*_10_}	{*f*_3_, *f*_7_, *f*_11_}	{*f*_4_, *f*_8_, *f*_12_}

**Table 7:** Ten cases of information values (bit) of the SHHS samples.

Cases	Reconstructed multiscale feature												Label
	SaO_2_ (%)				PR (BPM)				EEG (uV)				
	*f*_1_	*f*_2_	*f*_3_	*f*_4_	*f*_5_	*f*_6_	*f*_7_	*f*_8_	*f*_9_	*f*_10_	*f*_11_	*f*_12_	
X1	0.00093	0.00187	0.00280	0.00467	0.00435	0.00871	0.0131	0.0219	0.8533	1.2302	1.5895	1.8297	C1
X2	0.00102	0.00205	0.00308	0.00513	0.00515	0.01034	0.0115	0.0261	1.1602	1.2659	1.7055	1.9507	C1
X19	0	0	0.00019	0.00091	0.00086	0.00017	0.00026	0.00043	1.1168	1.3571	1.8146	2.2747	C2
X20	0	0	0	0	0.00222	0.00445	0.00669	0.01119	0.8437	1.0966	1.6145	2.1759	C1
X22	0.00017	0.00031	0.00043	0.00086	0.00479	0.0116	0.0177	0.0258	0.7982	1.0874	1.5239	2.1786	C1
X56	0.00024	0.00033	0.00054	0.00095	0.00258	0.00356	0.00455	0.00582	0.8321	1.1454	1.6789	2.3651	C2
X60	0.00096	0.00134	0.00245	0.00453	0.00189	0.00694	0.00781	0.00932	0.6809	1.0134	1.5706	2.0671	C2
X62	0	0	0	0	0.00094	0.00189	0.00284	0.00475	0.8087	0.9838	1.3175	1.8352	C2
X101	0.00172	0.00311	0.00544	0.0132	0.00255	0.00479	0.00682	0.0137	1.5772	2.0543	2.8914	2.6789	C1
X120	0.00293	0.00588	0.00886	0.01477	0.00383	0.00768	0.0115	0.0193	1.6334	2.1621	2.6054	2.7989	C1

**Table 8:** Different ranking results of feature selection algorithms.

Algorithm	Order of selected feature
IG	f3, f1, f2, f4, f5, f8, f9, f11, f12, f6, f7, f10
NMIFS	f2, f5, f3, f1, f6, f8, f4, f9, f10, f11, f12, f7
mRMR	f2, f3, f1, f8, f9, f10, f4, f5, f6, f12, f11, f7
MIFS-U	f2, f3, f1, f4, f5, f8, f9, f12, f6, f7, f11, f10
CR-MIFS	f2, f1, f3, f4, f5, f6, f9, f12, f7, f11, f8, f10

**Table 9:** Classification results were obtained with different methods.

Dataset	Author	Model	Structure	CV	Accuracy (a)%	F1-score	Kappa
Physionet SHHS	Arnaud et al. [47]	CNN	CNN	0.5/0.3/0/2	87.00	0.78	0.81
Physionet SHHS	Caihong et al. [48]	SleepContextNet	CNN + RNN	20	86.40	0.8	0.81
Physionet SHHS	Cong et al. [49]	XGBoost	\	5	87.5	\	0.81
Unpublished	Seda et al. [50]	K-NN and SVM	K-NN and SVM	10	92.78	0.93	\
Unpublished	Kuo et al. [51]	SNet	CNN	2	93.80	0.79	0.86
Physionet SHHS	Proposed scheme	SAPSO-SVM	SVM	5	97.22	0.94	0.88

## Data Availability

All the data in this manuscript are come from UCI machine learning repository
